# Exploring the Role of AI in Managing Treatment Recommendations for Lymphedema: International, Multidisciplinary, Multiprofessional Survey Study of Trust, Reliability, and Impact on Decision-Making

**DOI:** 10.2196/80553

**Published:** 2026-04-08

**Authors:** Adriano Fabi, Caroline E Egli, Séverin R Wendelspiess, Sebastian Griewing, Yvonne Haas, Laura De Pellegrin, Dirk J Schaefer, Shan S Qiu, Yves Harder, Elisabeth A Kappos

**Affiliations:** 1 Department of Plastic, Reconstructive, Aesthetic and Hand Surgery, University Hospital of Basel, Spitalstrasse 21, Basel, 4031, Switzerland, 41 613286254; 2 Faculty of Medicine, University of Basel, Spitalstrasse 21, Basel, Switzerland; 3 Institute for Digital Medicine, Philipps University of Marburg, Marburg, Germany; 4 Department of Gynecology and Obstetrics, Philipps University of Marburg, Marburg, Germany; 5 Department of Plastic and Hand Surgery, University Hospital of Bern, Bern, Switzerland; 6 Department of Plastic and Reconstructive Surgery, Maastricht University Medical Centre, Maastricht, The Netherlands; 7 Department of Plastic, Reconstructive and Aesthetic Surgery and Hand Surgery, University Hospital of Lausanne (CHUV), Lausanne, Switzerland; 8 Faculty of Biology and Medicine, University of Lausanne (UNIL), Lausanne, Switzerland; 9 Breast Center, University Hospital of Basel, Basel, Switzerland

**Keywords:** artificial intelligence, AI, ChatGPT, decision-making, digital health, large language models, lymphedema, personalized medicine

## Abstract

**Background:**

Upper and lower extremity lymphedema is a chronic, progressive condition that significantly impairs the quality of life of affected patients. Despite the recently established effectiveness of physical therapy and supermicrosurgical interventions, current guidelines frequently lag behind emerging evidence and commonly do not offer stage-specific treatment algorithms. This gap in evidence-based guidance may prompt clinicians with limited experience to seek support from large language models such as ChatGPT.

**Objective:**

Given the potential of artificial intelligence to rapidly integrate emerging research, this study evaluated how clinicians from different professional backgrounds rate the quality and reliability of personalized lymphedema management recommendations generated by ChatGPT.

**Methods:**

In this exploratory cross-sectional study, ChatGPT generated treatment recommendations for 6 standardized lymphedema case scenarios. An international panel of 67 participants (resident doctors, board-certified specialists, physiotherapists, and advanced practice nurses) from 34 institutions across 11 countries assessed the recommendations using a modified DISCERN questionnaire with a 9-point agreement scale ranging from 1 (completely disagree) to 9 (completely agree). Ratings were summarized as pooled means with variability measures and compared across clinician groups (residents vs board-certified physicians vs physiotherapists or advanced practice nurses) using group comparison testing.

**Results:**

ChatGPT was rated most favorably for diagnostic accuracy and treatment relevance, with higher ratings among residents than board-certified physicians. Residents assigned significantly lower scores for source indication, source currency, and communication of uncertainty. Between-group differences were observed across multiple DISCERN items, consistent with systematically more critical appraisal by experienced specialists. Participants reported moderate to high trust and willingness to consider ChatGPT as a supplementary resource, with more favorable perceptions among younger respondents.

**Conclusions:**

Clinicians perceived ChatGPT as potentially useful for preliminary orientation and educational support in lymphedema management, especially for less experienced users. Despite not being blinded, lower ratings in evidence transparency and uncertainty communication, particularly among experienced specialists, suggest that current artificial intelligence outputs should not be used as stand-alone guidance. Future work should test clinically integrated, citation-grounded workflows in prospective settings and evaluate whether they improve decision quality and efficiency.

## Introduction

Lymphedema is a chronic condition characterized by the accumulation of interstitial fluid, typically resulting from damage or disruption of lymphatic pathways [1]. In the Western world, lymphedema is commonly caused by oncologic surgery or radiotherapy and significantly reduces quality of life due to extremity swelling, limited mobility, and psychosocial distress [1-5].

Lymphedema treatment is highly individualized, influenced by age, preexisting comorbidities, and prior interventions [1]. Conservative treatment includes complex decongestive therapy; however, recent literature has demonstrated promising long-term outcomes after supermicrosurgical interventions, such as lymphovenous anastomosis (LVA) and vascularized lymph node transfer (VLNT) [6-9]. Due to the personalized nature of lymphedema treatment, following the current guidelines of the AWMF (Arbeitsgemeinschaft der Wissenschaftlichen Medizinischen Fachgesellschaften, S2k) or the International Society of Lymphology (ISL) may result in difficulties providing universally applicable, stage-specific recommendations [10,11]. Furthermore, the guidelines may include outdated information, primarily due to the time-intensive nature of expert consensus and validation processes [10,11]. Given the rapid advancements in lymphatic surgery, the incorporation of novel treatment approaches into official guidelines may take years, as this process requires a predefined and structured consensus. Consequently, there is a possibility that patients may receive care that is no longer up to date in a clinical context.

Large language models (LLMs) such as ChatGPT (OpenAI) represent a new approach to the processing of knowledge, which also applies to medical information [12,13]. These technologies have the potential to reshape medical decision-making by accelerating the integration of novel research into clinical routines [14-17]. Thus, unlike traditional guidelines, artificial intelligence (AI) is hypothesized to accelerate the transition of new findings into personalized treatment recommendations. However, whether this potential is truly realized in practice strongly depends on data implementation and prompting [18].

Therefore, the goal of this study was to assess ChatGPT’s ability to define and generate treatment recommendations for lymphedema by evaluating the quality, accuracy, and perceived utility of its recommendations across an interdisciplinary panel of relevant medical specialties. Additionally, the differences in acceptance were compared between resident doctors in training and board-certified plastic surgeons with experience in either lymphatic surgery or lymphedema management.

## Methods

### Study Design

The study was designed as a multidisciplinary assessment of ChatGPT-generated treatment recommendations of 6 cases of chronic lymphedema, ranging from ISL stage I to ISL stage 3 of the upper and lower limb (Multimedia Appendix 1). To ensure consistency, all cases were submitted to GPT-4o (GPT-4 Omni) on December 18, 2024, at noon Central European Time by the first authors (AF and CEE). Each case was entered into a distinct, separate chat session to limit session history bias. Only the initial response was considered, and the option to regenerate the response was not used. The responses obtained from GPT-4o were compiled into a PDF and were subsequently evaluated by a panel of resident doctors (considered inexperienced), board-certified doctors (considered experienced), and physiotherapists or advanced practice nurses (APNs) using a 9-point agreement scale [19].

### Clinical Vignettes and Treatment Recommendations

The clinical vignettes were designed with the intention of incorporating the underlying etiology of lymphedema through past medical history, current patient symptoms, and findings from clinical examination. Cases were prepared to be as realistic as possible and to reflect real clinical scenarios. Patients were clearly categorized into the 3 ISL stages, with corresponding indocyanine green studies illustrating the lymphatic patterns consistent with the stage described in each clinical vignette [10]. Furthermore, the cases were illustrated with images adapted from Principles and Practice of Lymphedema Surgery by Cheng et al [20]. A standardized prompt was used for each case and is provided in Multimedia Appendix 1.

### Evaluation

The evaluators had access to the original prompts, all 6 clinical cases, and the corresponding treatment recommendations generated by ChatGPT (Multimedia Appendix 1). The web-based questionnaire for subsequent evaluation was developed and distributed using the Research Electronic Data Capture (REDCap; Vanderbilt University) platform [21]. The DISCERN tool, a validated questionnaire for assessing the quality of written treatment information, was adapted to a 9-point agreement scale and then used for the quantitative evaluation of each generated response [19].

In accordance with the instructions provided in the DISCERN handbook [19], questions 1 and 2 (“Are the aims clear?” and “Does it achieve its aims?”) were excluded from the evaluation as they were found to be irrelevant to the defined research question of this study. Two additional questions were included in the evaluation of each case: one concerning correct staging (question 1) and the other regarding overall final agreement with the treatment option recommended by ChatGPT (question 16). In summary, each response was evaluated using 16 questions rated on a 9-point agreement scale, where 1 represents complete disagreement and 9 represents complete agreement. Finally, 3 concluding questions were added to evaluate the overall agreement regarding the treatment recommendation by ChatGPT and the attitude regarding AI use as a clinical decision tool (Multimedia Appendix 2). In addition, each participant was given the option to offer feedback. These open-text comments were reviewed descriptively by the first authors to provide contextual insight alongside the quantitative results. No formal qualitative analysis or coding approach was applied. All available comments were read in full, and relevant observations are summarized in Multimedia Appendix 3.

### Survey Distribution

The survey was conducted from January 7 to March 18, 2025. Resident doctors without prior experience in lymphedema management were recruited internationally through the same broader professional networks as the expert panel, primarily via participating centers in the LYMPH trial, as well as international resident and student networks. Experienced, board-certified doctors specializing in lymphedema treatment were identified and contacted, most of whom had previously participated in the international lymphedema consortium through existing collaborations [22]. A total of 64 board-certified doctors were invited via email to take part in the study, receiving a survey link along with a comprehensive explanation of its objectives and requirements (response rate 24/64, 37.5%). Additionally, 30 physiotherapists and lymphedema APNs specializing in lymphedema treatment were invited (response rate 10/30, 33.3%) through the same consortium and a contact list provided by the Lymphödem Vereinigung Schweiz (a Swiss patient organization) [22,23]. As resident recruitment was conducted via mass email and a preexisting student group, the response rate of invited resident doctors could not be calculated.

### Statistical Analysis

Statistical analysis was conducted using R software (version 4.4.3; R Foundation for Statistical Computing) [24]. The primary end point was the scoring of agreement and reliability of ChatGPT-generated treatment recommendations stratified by experience groups. Consequently, participants were categorized into 3 groups: resident doctors, board-certified doctors, and physiotherapists or APNs. For each question, group-wise means and SDs were calculated. The Levene test was used to confirm homogeneity of variances prior to conducting 1-way ANOVA. Depending on the result of the Levene test, either standard ANOVA (for equal variances) or Welch ANOVA (for unequal variances) was used to test for differences between groups. Where applicable, an “overall” group comprising all participants was included for descriptive purposes only and was excluded from inferential statistical testing. A 2-sided *P* value of ≤.05 was considered statistically significant.

### Ethical Considerations

This study was conducted in accordance with the Declaration of Helsinki. On the basis of the fictional nature of the cases and the absence of human subject data, the local ethics committee (Ethikkommission Nordwest- und Zentralschweiz) deemed that no formal ethics approval was required. Participation in the web-based survey was voluntary. All participants were informed about the purpose of the study, and informed consent was implied through completion of the survey. Participants had the option to discontinue the survey at any time without any consequences. All data were collected anonymously. Data protection and confidentiality were ensured in accordance with applicable regulations. No compensation was provided. No identifiable individuals are shown in any figures or supplementary materials.

## Results

A total of 67 participants from 34 different institutions across 11 countries completed the survey, including 33 (49.3%) resident doctors, 24 (35.8%) board-certified doctors, and 10 (14.9%) lymphedema physiotherapists or APNs. The mean age was 35.6 (SD 14.1) years, with resident doctors averaging 28.0 (SD 6.0) years, board-certified doctors 45.0 (SD 14.0) years, and physiotherapists or APNs 42.0 (SD 19.9) years.

### Diagnostic Accuracy and Relevance (Q1 and Q2) 

**Figure 1.**
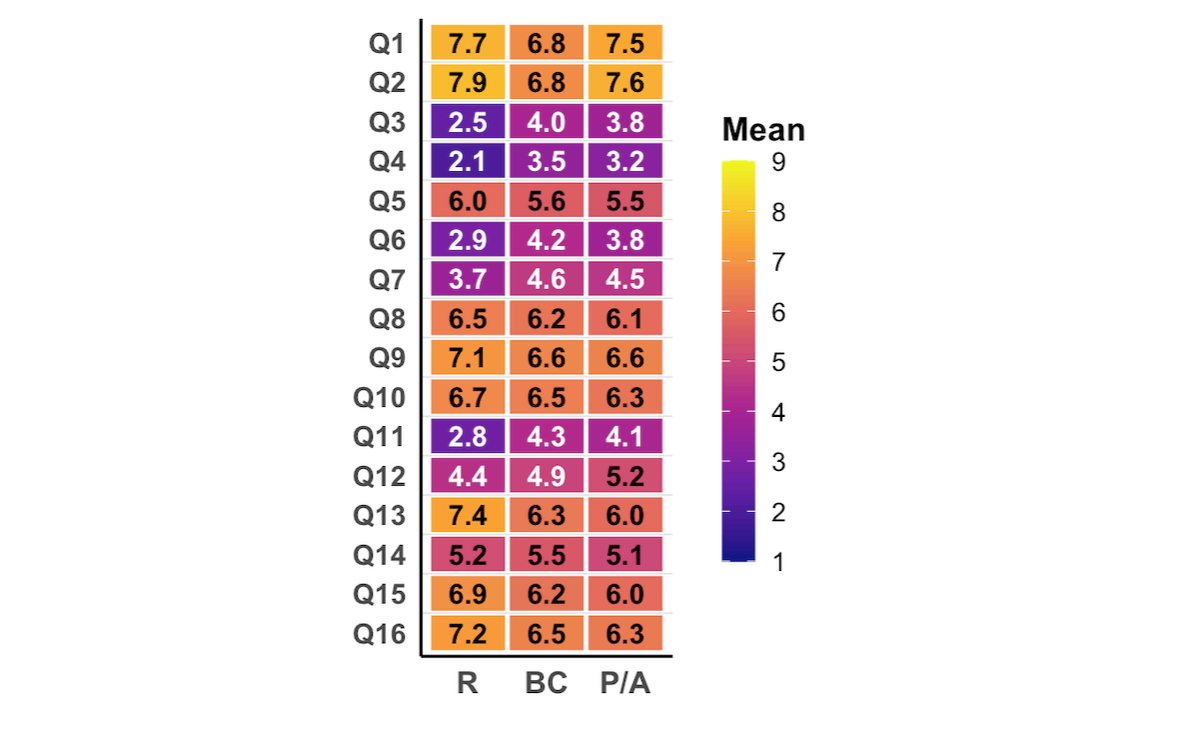
Heat map of pooled mean item ratings across 6 case scenarios by clinician group. Tiles are color-coded by the pooled mean score for each question (Q1-Q16) within each group; numeric values within tiles indicate the corresponding mean. Higher scores indicate more favorable ratings on a 9-point agreement scale (1=completely disagree and 9=completely agree); variability measures (SD or CI) are not shown in this figure. BC: board-certified; R: resident; P/A: physiotherapist/advanced practice nurse.

Staging and diagnostic accuracy (question 1; Q1) were generally rated high (mean 7.3, SD 1.5), with board-certified doctors assigning significantly lower scores than resident doctors (*P*<.001; Table 1). Similarly, the relevance of the recommendations (Q2) was rated highly (mean 7.5, SD 1.4), with board-certified doctors rating significantly lower compared to resident doctors or physiotherapists or APNs (*P*<.001; Figure 1; Table 1). A detailed overview of scoring per case is provided in Multimedia Appendix 4.

**Table 1.** Mean scores across all 6 scenarios^a^.

Question^b^	Overall (n=67), mean (SD)	Resident doctors (n=33), mean (SD)	Board-certified physicians (n=24), mean (SD)	Physiotherapist or APN^c^ (n=10), mean (SD)	*P* value
Q1—diagnostic accuracy	7.3 (1.5)	7.7 (1.2)	6.8 (1.6)	7.5 (1.9)	*<.001^d^*
Q2—relevance	7.5 (1.4)	7.9 (1.0)	6.8 (1.6)	7.6 (1.1)	*<.001*
Q3—indication of sources used	3.2 (2.5)	2.5 (2.1)	4.0 (2.4)	3.8 (2.9)	*<.001*
Q4—currency of sources used	2.7 (2.1)	2.1 (1.6)	3.5 (2.1)	3.2 (2.9)	*<.001*
Q5—balanced and unbiased information	5.8 (1.8)	6.0 (1.7)	5.6 (1.6)	5.5 (2.2)	.07
Q6—additional supporting information	3.5 (2.3)	2.9 (2.1)	4.2 (2.0)	3.8 (2.7)	*<.001*
Q7—communication of areas of uncertainty	4.1 (2.1)	3.7 (2.0)	4.6 (1.8)	4.5 (2.5)	*<.001*
Q8—explanation of how the treatment works	6.3 (1.8)	6.5 (2.0)	6.2 (1.5)	6.1 (2.0)	.23
Q9—benefits of treatment	6.8 (1.6)	7.1 (1.8)	6.6 (1.3)	6.6 (1.7)	*.04*
Q10—risks of treatment	6.6 (1.9)	6.7 (2.0)	6.5 (1.5)	6.3 (2.0)	.22
Q11—consequences of treatment refusal	3.5 (2.2)	2.8 (2.0)	4.3 (1.9)	4.1 (2.7)	*<.001*
Q12—impact on quality of life	4.7 (2.4)	4.4 (2.3)	4.9 (2.1)	5.2 (2.8)	*.03*
Q13—availability of multiple treatment options	6.8 (1.9)	7.4 (1.7)	6.3 (1.8)	6.0 (2.2)	*<.001*
Q14—support for shared decision-making	5.3 (2.3)	5.2 (2.4)	5.5 (2.1)	5.1 (2.3)	.37
Q15—overall quality of the recommendation	6.5 (1.6)	6.9 (1.4)	6.2 (1.8)	6.0 (1.8)	*<.001*
Q16—overall agreement	6.8 (1.8)	7.2 (1.6)	6.5 (1.9)	6.3 (2.3)	*<.001*

a Values represent the pooled mean (SD) across the 6 standardized lymphedema case scenarios from an exploratory cross-sectional survey of 67 clinicians (resident doctors, board-certified physicians, and physiotherapists or advanced practice nurses) from 34 institutions in 11 countries (January-March 2025).

b Q: question.

c APN: advanced practice nurse.

d Values in italic demonstrate statistical significance (*P*<.05).

### Source Transparency and Objectivity (Q3-Q5) 

**Figure 2.**
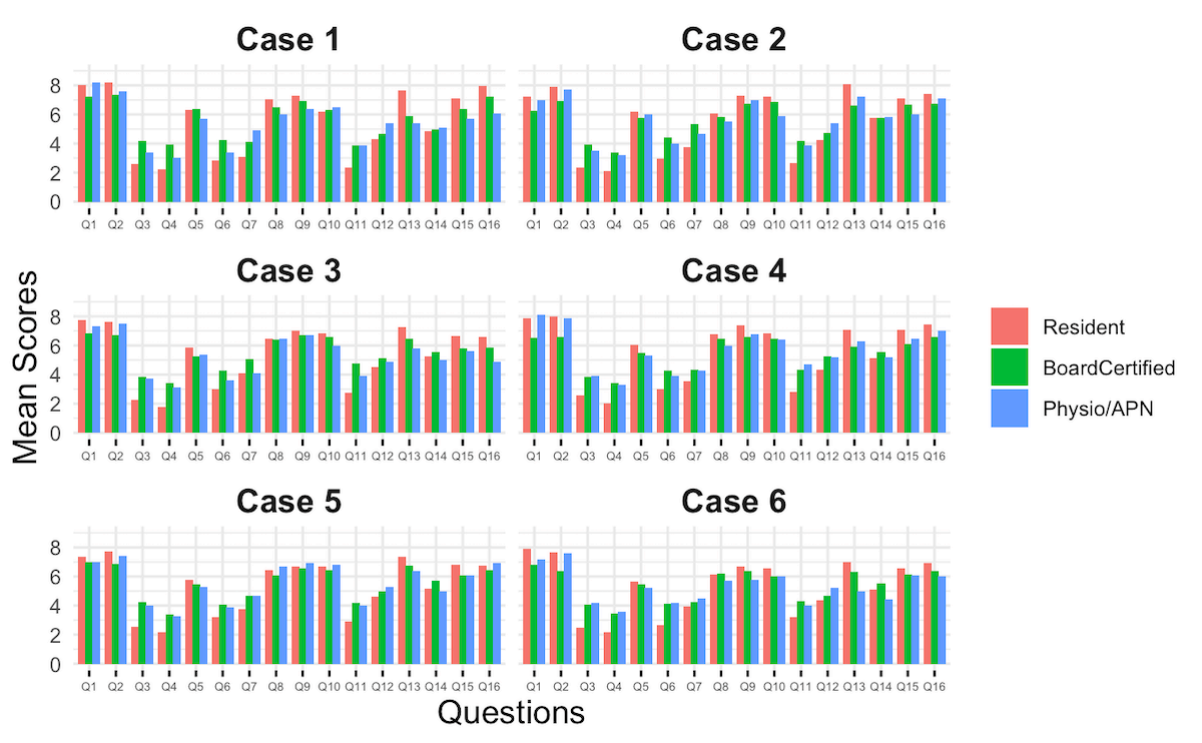
Mean scores by professional group for all cases. Overview of mean scores across 6 lymphedema case scenarios by professional group. Bars represent mean scores. APN: advanced practice nurse; Q: question. Exact values can be found in Multimedia Appendix 4.

The clarity of source citation (Q3) and the dating of sources (Q4) were rated very low across all groups, with mean scores of 3.2 (SD 2.5) and 2.7 (SD 2.1), respectively (Figure 2). Interestingly, resident doctors gave both questions significantly lower ratings than the other groups (*P*<.001). Interestingly, Q5 (objectivity or neutrality of ChatGPT) received an average score of 5.8 (SD 1.8), with no significant differences between groups.

### Supportive Information and Areas of Uncertainty (Q6 and Q7)

The quality of additional resources (Q6) was rated badly, with a mean score of 3.5 (SD 2.3) across all cohorts. Similarly, the communication of uncertainty (Q7) received moderate scores of 4.1 (SD 2.1), with residents giving the lowest scores across both questions (*P*<.001).

### Treatment Explanation and Options, Risk Communication, and Quality of Life Considerations (Q8-Q13)

ChatGPT’s performance in communicating treatment options and possible risks was generally rated positively, with Q8 (how the treatment would work), Q9 (benefits of treatment), and Q10 (potential risks) all receiving favorable scores (mean 6.3, SD 1.8; mean 6.8, SD 1.6; and mean 6.6, SD 1.9, respectively). In contrast, Q11 (consequence of no treatment) consistently scored low (mean 3.5, SD 2.2). Q12 assessed whether the provided information addressed the impact of the treatment on patients’ quality of life. Ratings were moderate (mean 4.7, SD 2.4), with residents rating the responses significantly lower than other groups. Q13 (ChatGPT mentioning that there may be more than one treatment choice) was generally rated positively (mean 6.8, SD 1.9).

### Shared Decision-Making and Overall Quality (Q14-Q16)

Support for shared decision-making (Q14) was rated moderately (mean 5.3, SD 2.3). The overall impression of the quality of responses (Q15) and overall agreement with treatment recommendations (Q16) both received high ratings (mean 6.5, SD 1.6 and mean 6.8, SD 1.8, respectively), with physiotherapists or APNs rating the answers the lowest (*P*<.001).

### Concluding Questions—Trust and Future Use

**Figure 3.**
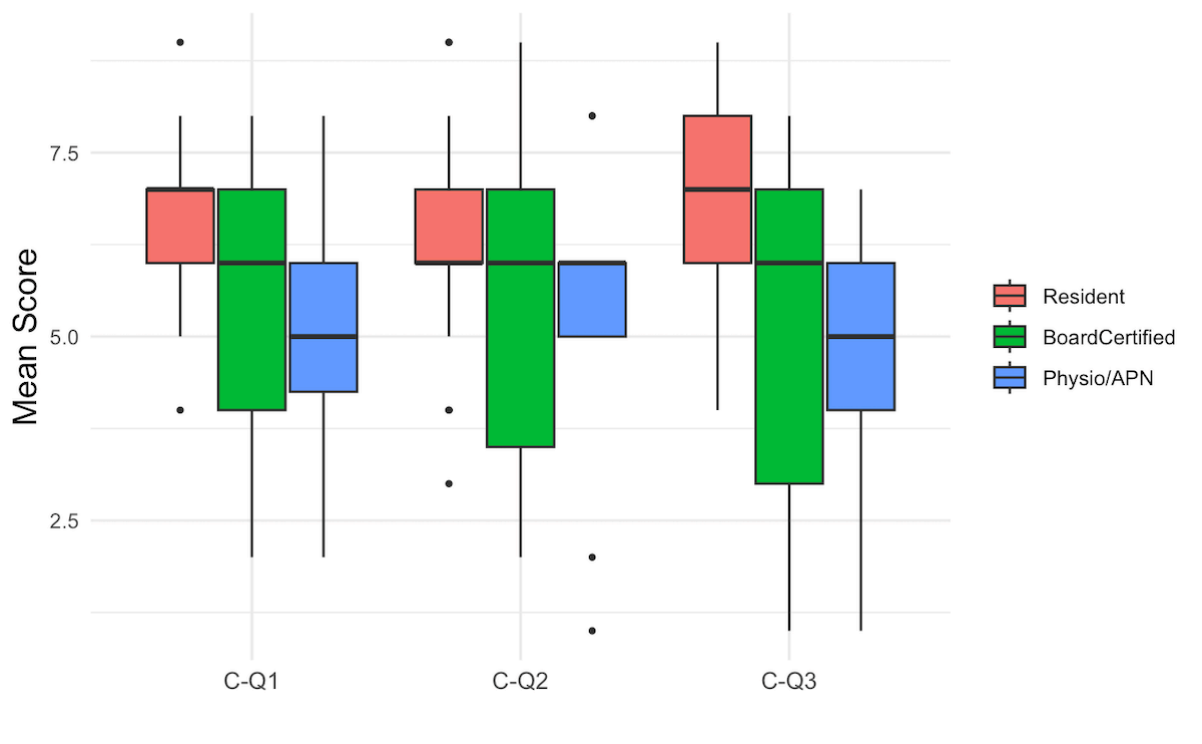
Concluding questions (CQs) by professional groups. Boxplot overview of concluding questions (trust, willingness to use, and recommendation of ChatGPT) by professional group. APN: advanced practice nurse.

Concluding question (CQ) 1 assessed trust in ChatGPT’s responses, CQ2 assessed willingness to use ChatGPT in future clinical work, and CQ3 assessed whether participants would recommend it for clinical decision-making. Resident doctors rated all 3 concluding questions (CQ1-CQ3) significantly higher than board-certified doctors and physiotherapists or APNs. CQ2 showed a similar pattern, with resident doctors at a mean of 6.5 (SD 1.4), board-certified doctors at 5.3 (SD 2.2), and physiotherapists or APNs at 5.3 (SD 2.3). Finally, for CQ3, which assessed whether participants would recommend AI as a clinical decision-making tool, residents again scored highest, followed by board-certified doctors and physiotherapists or APNs (Figure 3; Table 2). All survey comments are listed in Multimedia Appendix 3.

**Table 2.** Scores by professional groups across all concluding questions (CQs)^a^.

Question	Overall (n=67), mean (SD)	Resident doctors (n=33), mean (SD)	Board-certified physicians (n=24), mean (SD)	Physiotherapist or APN^b^ (n=10), mean (SD)	*F* test (*df*)	Levene test for equality of variables	ANOVA *P* value
CQ1—trust in AI^c^	5.9 (1.5)	6.5 (1.0)	5.4 (1.9)	5.1 (1.8)	5.21 (2, 20.58)	0.027	*.007^d^*
CQ2—willingness to use AI	5.9 (1.9)	6.5 (1.4)	5.3 (2.2)	5.3 (2.3)	3.20 (2, 63.00)	0.119	*.04*
CQ3—recommendation of AI	6.1 (2.1)	7.0 (1.4)	5.4 (2.4)	4.7 (2.0)	8.13 (2, 21.55)	0.015	*<.001*

a Mean scores for the CQS (trust, willingness to use, and recommendation of ChatGPT), stratified by professional group.

b APN: advanced practice nurse.

c AI: artificial intelligence.

d Values in italics were statistically significant (*P*<.05).

## Discussion

In this exploratory, cross-sectional study, a total of 67 participants, comprising 33 resident doctors still in training, 24 board-certified doctors, and 10 lymphedema physiotherapists or APNs, from 34 different institutions across 11 countries evaluated ChatGPT-generated treatment recommendations for 6 fictional lymphedema cases. This is the first study to evaluate ChatGPT-generated treatment recommendations for lymphedema.

### Notably Higher Acceptance of ChatGPT Among Younger Doctors

The overall high level of agreement with the ChatGPT-generated treatment recommendations (Q16) indicates broad acceptance across all cases and groups (Table 1). Resident doctors expressed a more positive opinion toward ChatGPT, a tendency that is presumably attributable to their lesser clinical experience and greater openness to digital technologies in medical practice, indicating that the evaluation of ChatGPT may also be influenced by generational factors (Figure 3) [25,26]. Importantly, however, this favorable perception was not uncritical. Residents assigned significantly lower scores than board-certified physicians for source transparency (Q3-Q4; Figure 1). The lower ratings may reflect stricter expectations regarding explicit source citation, differences in grading thresholds, or a reduced ability to recognize implicit sources that experienced clinicians may infer based on prior knowledge [27]. As the study design does not allow discrimination between these explanations, no conclusions can be drawn regarding the underlying cause of this difference. Nevertheless, several resident doctors reported that ChatGPT already serves them as a useful orientation tool, particularly in situations with limited prior familiarity with a condition or treatment options. This underscores the tool’s potential as a rapid decision support or educational aid, as it only uses a few seconds to generate an extensive response [18]. It also highlights the need to integrate the critical use of AI tools into medical education. As residents showed greater willingness to use ChatGPT, targeted training could help prevent misconceptions and ensure these tools complement, rather than replace, clinical reasoning.

However, board-certified doctors demonstrated a lower level of agreement with treatment recommendations provided by ChatGPT, possibly due to their greater clinical experience and higher expectations regarding therapeutic nuances. This may, in part, stem from the perception that AI systems are not yet capable of providing fully personalized treatment algorithms. This viewpoint was supported in the open comments section (Multimedia Appendix 3), where experienced clinicians repeatedly pointed out that well-established surgical interventions, such as LVA and VLNT, were insufficiently addressed. In this study, LVA and VLNT were only briefly mentioned in cases 2, 3, 4, and 6 and only as potential options if compression therapy and liposuction were deemed insufficient. Reconstructive procedures such as LVA and VLNT are mentioned in both the German AWMF (S2k) guidelines and the ISL consensus document [10,11]. While the AWMF (S2k) guidelines suggest considering surgical options only after 6 months of conservative treatment without any lasting beneficial effect, the ISL guidelines refer to microsurgical interventions without specifying any time frame regarding previous complex decongestive therapy [10,11]. However, recent literature suggests that these procedures may also be used earlier or even as a preventative measure, thus challenging the traditional time threshold [28,29]. Given that key papers and guideline discussions on LVA and VLNT were already available well before October 2023, their limited presence in the responses is unlikely to be explained by the model’s knowledge cutoff alone [30]. A more plausible explanation is that ChatGPT defaults to what it has seen most often: conservative management pathways that are widely taught and described, and a generally “play it safe” tendency when asked broad clinical questions [31]. In other words, the model may not be unaware of surgery; rather, it may just not surface it unless the prompt explicitly asks for surgical options or provides more detail about operative candidacy [12]. Nevertheless, newer ideas such as prophylactic LVA may still be underrepresented due to the recency of the evidence. Future work should test whether prompting specifically for surgical options changes the balance and completeness of the recommendations. These findings also emphasize the necessity of incorporating temporal limitations when using AI tools in clinical decision-making processes [7,8,20,28-30,32,33]. Furthermore, the strong emphasis on lymphedema management using conservative treatment options may result from the broader and more established evidence base in this area, which has developed over decades and is therefore more prominently represented in ChatGPT’s training data.

Physiotherapists or APNs expressed greater skepticism regarding ChatGPT’s treatment recommendations, as highlighted in the open comments section. The recommendations were regarded as being too generalized and lacking personalization. It was emphasized that effective lymphedema care necessitates ongoing, individualized assessment, a component that the AI recommendations did not adequately address.

### Prompt Quality and Lack of Sources Limit ChatGPT’s Clinical Reliability

One of the key characteristics of ChatGPT is its reliance on user input—responses are generated solely based on the prompt it receives [12]. As a result, the specificity and clarity of the input significantly affect the quality, depth, and relevance of the output [12]. Shorter prompts tend to produce shorter, less detailed responses. Citations were completely absent in ChatGPT’s responses and consistently rated poorly by participants (Q3; Table 1), demonstrating that the absence of sources significantly limited the reliability of AI-generated treatment recommendation and, consequently, did not earn the physicians’ trust. Furthermore, the absence of information regarding additional sources (Q6), the lack of mention of areas of uncertainty (Q7), and the omission of potential consequences of forgoing treatment (Q11) were also rated negatively (Table 1).

As an LLM, ChatGPT does not inherently verify the information it provides, but instead generates content based on linguistic patterns, which may influence the accuracy of treatment recommendations [12]. Notably, previous studies emphasized the need to explicitly ask for citations, as the model may otherwise omit references or produce so-called “hallucinations,” meaning fabricated or incorrect references [34,35]. As our prompt did not explicitly request sources, thus reflecting the expected real-world use of ChatGPT, the absence of citations was expected. However, this highlights 2 key limitations. First, when users lack the background knowledge to formulate precise prompts, the model’s effectiveness is significantly reduced. Second, users currently have no possibilities to verify the origin or quality of the information provided. Previous studies have shown that such uncritical reliance on unverifiable information has the potential to result in inefficient or inappropriate treatment decisions and, consequently, potentially harm the patient [36]. It is also important to emphasize that ChatGPT does not *collect* information but rather generates responses based on statistical patterns in its training data, which further limit its interpretive reliability.

ChatGPT may also pose security risks [12]. Of particular concern is the use of false or deliberately misleading prompts, the aim of which is to manipulate the model into generating inaccurate responses [12]. To ensure patient safety, any clinical implementation would require strict regulatory oversight. A more realistic, and potentially safer, alternative could involve embedding ChatGPT within a clinical decision support system using prevalidated clinical data [37]. In this scenario, ChatGPT would function as a predictive model, assisting in decision-making within a strictly regulated and supervised data environment.

### Study Strengths and Limitations

This is the first exploratory, cross-sectional study evaluating the agreement, reliability, and decision-making value of AI using LLMs in generating treatment recommendations for lymphedema management in a global, multidisciplinary, and interprofessional panel. Strengths of this study include the sizeable expert panel of 67 doctors and lymphedema therapists from 34 different institutions across 11 countries. A potential limitation of this study is that evaluators were aware that responses were generated by ChatGPT, which may have introduced either positive bias (novelty or expectation effect) or negative bias (preexisting skepticism toward AI). The results should therefore be interpreted as clinicians’ perceptions of AI-generated recommendations rather than a purely objective measure of model performance. The lack of human-generated reference standard represents an important limitation, as it prevents the interpretation of absolute DISCERN scores; these should instead be understood in a relative context across professional groups. Although the 6 vignettes were deliberately designed to reflect common clinical presentations across the ISL stage spectrum across both extremities, they cannot capture the full heterogeneity of lymphedema. Consequently, generalizability to all clinical contexts is limited, and future studies should evaluate a larger and more diverse set of cases. Another limitation of this study is the use of an adapted assessment tool based on the DISCERN criteria. Although the underlying framework is validated, modifications to the item set and scoring scale may have altered the original psychometric properties of the instrument. As resident recruitment was conducted via large email and international student group, the response rate of invited resident doctors could not be calculated. Moreover, all analyses were based on a single, time-stamped response per case and were not controlled for random seed values or multirun variability, as this would have substantially increased survey burden. Furthermore, because the study was conducted using the standard ChatGPT web interface rather than the application programming interface, we were unable to access or control key inference parameters such as temperature, top-p, or system prompts. As a result, these factors were not reported. Finally, the study did not assess how variations in prompt design might affect the consistency or accuracy of the generated recommendations. Additionally, participants did not generate the prompts themselves, which may limit user satisfaction to the generated responses.

### Conclusions

ChatGPT has the capacity to provide relevant basic knowledge for lymphedema management and may be particularly useful for less experienced clinicians. However, it has not yet succeeded in replacing expert consultation and offers only limited personalization. Additionally, its constraints regarding source transparency and prompt dependency highlight the need for cautious and context-aware integration into clinical practice.

## Supplementary material

10.2196/80553 Multimedia Appendix 1 Prompt, cases, and ChatGPT treatment recommendations.

10.2196/80553 Multimedia Appendix 2 Research Electronic Data Capture questionnaire with the validated DISCERN tool.

10.2196/80553 Multimedia Appendix 3 Survey comments per subgroup (anonymous).

10.2196/80553 Multimedia Appendix 4 Group comparisons for questionnaire responses divided by case.

## Abbreviations

AI: artificial intelligence
APN: advanced practice nurse
AWMF: Arbeitsgemeinschaft der Wissenschaftlichen Medizinischen Fachgesellschaften
ISL: International Society of Lymphology
LLM: large language model
LVA: lymphovenous anastomosis
REDCap: Research Electronic Data Capture
VLNT: vascularized lymph node transfer
