# Supplementary material for: Exploring the Role of AI in Managing Treatment Recommendations for Lymphedema: International, Multidisciplinary, Multiprofessional Survey Study of Trust, Reliability, and Impact on Decision-Making
Source: JMIR Med Inform. 2026 Apr 8;14:e80553. doi: 10.2196/80553 (PMC13060743; doi:10.2196/80553)
Supplement: Multimedia Appendix 1 [file medinform-v14-e80553-s001.docx]

**Supplementary Material 1.** Prompt, Cases & ChatGPT Treatment Recommendations

***Case Prompt:***

*“*Using the clinical vignette and photo documentation provided, make a diagnosis and recommend an evidence-based treatment plan. Provide a brief rationale for the most optimal therapy, including benefits and associated risks.”

***Case 1: Mild Upper Extremity Lymphedema***

A 50-year-old female presents with a 1-year history of mild, intermittent swelling of the left hand, fingers, and forearm (left: 24.5 cm vs. right: 24.3 cm), with pitting edema resolving upon elevation. The patient has a history of triple-negative breast cancer of the left breast treated with oncoplastic tumorectomy, axillary surgery and adjuvant radiochemotherapy. No pain, restricted motion, systemic symptoms, lymphadenopathy, or disease recurrence. Indocyanine Green Lymphography shows patent lymphatics without dermal backflow.”

*ChatGPT Treatment Recommendation: (11 Seconds)*

**Diagnosis:**  Early-stage (Stage I) secondary lymphedema of the left upper extremity following breast cancer treatment.

**Rationale for Diagnosis:**
The patient’s presentation - mild, intermittent swelling that is pitting and improves with limb elevation - is consistent with early-stage lymphedema. This aligns with post-breast cancer surgery and radiation changes in the lymphatic drainage. The absence of pain, joint restriction, systemic symptoms, and disease recurrence, coupled with the Indocyanine Green (ICG) lymphography showing patent lymphatics and no dermal backflow, supports a diagnosis of early or Stage I lymphedema rather than more advanced disease.

**Recommended Treatment Plan:**

1. Compression Therapy:
   - Graduated Compression Sleeves or Gauntlets: Wearing a well-fitted compression garment during the day helps reduce edema by promoting venous and lymphatic return.
2. Manual Lymphatic Drainage (MLD):
   - A specialized form of gentle massage performed by a certified therapist can help mobilize interstitial fluid into functioning lymphatic channels.
3. Exercise and Physical Activity:
   - Supervised, low-intensity exercise (e.g., light resistance training, stretching, yoga): Facilitates muscle pumping to improve lymphatic and venous return.
   - Regular physical activity also helps maintain overall limb mobility and function.
4. Skin Care and Hygiene:
   - Keeping the skin clean, well-hydrated, and protected reduces the risk of infection (cellulitis) that can exacerbate lymphedema.
5. Lifestyle Modifications:
   - Elevation of the affected limb when resting, avoiding constrictive clothing or jewelry, and maintaining a healthy body weight.

**Rationale for Recommended Therapy:**

- Benefits:
  - These conservative measures have strong evidence for slowing disease progression, reducing edema, and improving quality of life. Compression garments and MLD have long-standing support in clinical guidelines as first-line management for early-stage lymphedema. Exercise has been shown to be both safe and beneficial, enhancing lymphatic flow without increasing risk.
- Risks and Considerations:
  - Minimal Risks: Properly fitted compression garments and gentle MLD therapy carry low risk. Some patients may experience mild discomfort or difficulty with adherence, but professional guidance helps minimize these issues.
  - Infection Risk: While infections are rare with appropriate skincare, untreated infections can worsen lymphedema. Therefore, proper hygiene and prompt treatment of any skin changes are essential.

In summary, an evidence-based conservative management approach — compression, manual lymph drainage, exercise, and skin care — provides the best balance of efficacy and safety for early-stage lymphedema.

***Case 2: Moderate Upper Extremity Lymphedema***

“A 50-year-old female presents with a 3-year history of moderate, swelling of the left hand, fingers, and forearm (25.6 cm vs. 24.3 cm), with pitting edema and no resolution upon elevation. The patient has a history of breast-conserving surgery and adjuvant radiochemotherapy for an adenocarcinoma (R0). Sentinel lymph node biopsy was negative. She reports recurrent pain, reduced range of motion, and a cellulitis episode 3 months ago, which was resolved with antibiotics and compression therapy. Indocyanine Green Lymphography shows splash patterns near the axilla and patent lymphatic vessels.”

*ChatGPT Treatment Recommendation: (11 Seconds)*

**Diagnosis:** Secondary (breast cancer–related) lymphedema of the upper extremity.

**Evidence-Based Treatment Plan:**

1. Complete Decongestive Therapy (CDT): This includes manual lymphatic drainage (MLD), use of compression bandaging or garments, meticulous skin care, and a tailored exercise regimen to optimize lymphatic flow.
2. Adjunctive Measures:
   - Continue wearing well-fitted compression sleeves or garments, especially during waking hours and physical activity.
   - Implement a supervised exercise program focusing on gentle range-of-motion exercises, resistive exercises with gradual progression, and aerobic activities to stimulate lymphatic return.
   - Maintain good skin hygiene to reduce the risk of recurrent cellulitis.
3. Consideration of Supermicrosurgical Intervention (LVA): If conservative measures are insufficient, lymphaticovenous anastomosis (LVA) may be considered. The presence of patent lymphatic vessels demonstrated by indocyanine green lymphography suggests potential benefit from minimally invasive, microsurgical techniques aimed at improving lymphatic drainage.

**Rationale for the Most Optimal Therapy:**
Complete Decongestive Therapy (CDT) is widely regarded as the first-line, gold-standard, conservative management for secondary lymphedema. It addresses fluid accumulation, improves limb circumference and range of motion, decreases discomfort, and helps prevent complications like recurrent cellulitis.

- Benefits:
  - Noninvasive and well-established efficacy.
  - Improves limb volume and mobility.
  - Reduces infection risk through better edema control and skin care.
- Risks/Considerations:
  - Time-intensive and requires patient adherence for long-term success.
  - May need periodic garment refitting and ongoing professional support.

**Surgical Interventions (LVA):**

- Benefits:
  - Potential for improved, longer-lasting volume reduction.
  - May decrease the frequency of cellulitis episodes by improving lymphatic drainage.
- Risks:
  - As a surgical procedure, it carries the standard surgical risks (infection, bleeding, and anesthesia-related risks).
  - Outcomes may vary, and success depends on adequate preoperative imaging and surgical expertise.

In summary, starting with CDT and adjunctive conservative measures is the most supported evidence-based approach. Should conservative measures plateau, LVA can be considered as an additional, minimally invasive surgical option to further improve long-term outcomes.

***Case 3: Advanced Upper Extremity Lymphedema***

“A 50-year-old female presents with an eight-year history of severe and persistent non-pitting swelling of the left hand, fingers, and arm (25.9 cm vs. 24.3 cm), with skin fibrosis and hyperkeratosis. The patient has a history of breast-conserving surgery, radical axillary lymph node dissection, and radiochemotherapy for an adenocarcinoma of the left breast (R0). She reports constant pain, reduced range of motion, and recurrent erysipelas and cellulitis requiring antibiotics. Compression therapy and manual lymphatic drainage provide only minimal, short-term relief. Indocyanine Green Lymphography shows a stardust pattern indicating lymphatic scarring.”

*ChatGPT Treatment Recommendation: (18 Seconds)*

**Diagnosis:** The clinical picture is consistent with advanced, chronic lymphedema of the left upper extremity secondary to breast cancer treatment (breast-conserving surgery, radical axillary lymph node dissection, and radiochemotherapy). The persistent non-pitting edema, skin fibrosis, hyperkeratosis, recurrent infections, and “stardust” pattern on Indocyanine Green (ICG) lymphography, all support a diagnosis of severe, lymphatic flow–impaired lymphedema.

**Recommended Evidence-Based Treatment Plan:**

1. Surgical Intervention (Liposuction): Liposuction of the affected limb to remove the excess fibroadipose tissue that has accumulated due to long-standing lymphedema. While it does not restore lymphatic flow, it effectively reduces limb volume and improves limb contour.
2. Postoperative and Long-Term Compression Therapy: Continuous, high-quality compression garments worn daily to maintain the volume reduction achieved by liposuction and to help prevent recurrent swelling.
3. Adjunctive Measures:
   - Complete Decongestive Therapy (CDT): Even though the patient has had limited benefit previously, periodic sessions of manual lymphatic drainage, meticulous skin care, and targeted range-of-motion exercises remain essential to maintain skin integrity, reduce the risk of infections, and preserve joint mobility.
   - Physical Therapy and Exercise Programs: Supervised exercises may help improve lymphatic pumping and overall function.
   - Consideration of Lymphatic Microsurgical Procedures: In select cases where some functional lymphatic vessels remain (assessed via ICG and lymphoscintigraphy), lymphaticovenular anastomosis (LVA) or vascularized lymph node transfer (VLNT) may be considered to restore some lymphatic flow. These are more complex and have varied success rates, but may be beneficial in carefully selected patients after volume reduction.

**Rationale for the Optimal Therapy (Liposuction and Compression):**

Benefits:

- - Significant Volume Reduction: Liposuction has been well-documented to yield substantial and persistent volume reduction in advanced lymphedema, improving limb shape, reducing pain, and enhancing quality of life.
  - Improved Mobility and Function: By decreasing the weight and bulk of the limb, patients often experience better range of motion and reduced discomfort.
  - Decreased Infection Rates: Lower limb volume can reduce the frequency of bacterial entry points in thickened, fibrotic skin, thereby lowering the incidence of cellulitis and erysipelas.

Risks and Considerations:

- - Surgical and Anesthetic Risks: As with any surgical procedure, liposuction carries risks such as bleeding, infection, and anesthesia-related complications.
  - Need for Lifelong Compression: The positive effects of liposuction are maintained only if the patient adheres to strict compression therapy indefinitely. Without compression, the limb volume may gradually return.
  - Limited Restoration of True Lymphatic Function: Liposuction does not inherently restore lymphatic drainage pathways. It improves symptoms by removing excess tissue rather than correcting the underlying lymphatic dysfunction. In highly advanced disease, microsurgical interventions (LVA, VLNT) have variable success and may be considered adjunctively.

In summary, for a patient with advanced, fibrotic lymphedema unresponsive to conservative measures, liposuction followed by rigorous compression therapy is an evidence-based, reliable method to achieve sustained volume reduction, symptom relief, and improved quality of life.

***Case 4: Idiopathic Mild Lower Extremity Lymphedema***

“A 50-year-old female patient presents with a one-year history of mild, intermittent minimal swelling of the left foot and lower leg (37.1 cm vs. 36.9 cm) with pitting edema resolving upon elevation. The patient is in good general health, with no history of cancer, trauma, or infection. No pain or restricted motion. Indocyanine Green Lymphography shows patent lymphatic vessels and no dermal backflow.”

*ChatGPT Treatment Recommendation: (16 Seconds)*

**Diagnosis:** Early-stage (Stage I) primary lymphedema of the lower extremity.

**Rationale for Diagnosis:**

- The patient presents with intermittent, mild swelling of the foot and lower leg that is minimal in circumference difference and pitting in nature.
- Swelling that improves with elevation is consistent with early lymphedema, as fluid accumulation is not yet severe or fibrotic.
- The absence of a history of cancer, trauma, or infection reduces the likelihood of secondary lymphedema.
- The Indocyanine Green (ICG) lymphography showing patent lymphatic vessels and no dermal backflow suggests that lymphatic drainage pathways remain functional, aligning with early changes seen in Stage I lymphedema, where there are subtle abnormalities but no significant irreversible changes.

**Evidence-Based Treatment Plan:**

1. Compression Therapy: Use of properly fitted, graduated compression stockings during the day to facilitate fluid return and prevent progression of swelling.
2. Exercise and Mobility: Encouragement of low-impact, regular exercises (e.g., walking, cycling, light resistance training) to enhance muscle pump action and lymphatic flow.
3. Manual Lymphatic Drainage (MLD) if Needed: Although not mandatory at this stage, occasional MLD by a trained therapist can help optimize lymph flow.
4. Skin Care: Gentle skin care routines to maintain skin integrity and prevent infections (e.g., moisturizing to avoid dryness and cracks, prompt treatment of any skin irritation).

**Brief Rationale for Optimal Therapy:**

- Benefits: Compression therapy and exercise are first-line, non-invasive measures with a strong evidence base for managing early lymphedema. They help maintain limb volume, improve comfort, and potentially halt progression to more advanced disease stages. This conservative approach is cost-effective, safe, and generally well-tolerated.
- Risks: Properly fitted compression garments have minimal risks, though they may cause discomfort if not sized correctly. Skin irritation or breakdown is possible but rare with proper hygiene and garment care. Overall, the chosen treatments carry low risk with substantial potential benefit in preventing further lymphatic compromise.

***Case 5: Moderate Lower Extremity Lymphedema***

“A 50-year-old female presents with a three-year history of moderate, constant swelling of the left foot and lower leg (38.2 cm vs. 36.9 cm), with pitting edema and no resolution upon elevation. The patient has a history of bilateral adnexectomy, pelvic and paraaortal lymphadenectomy for a well-differentiated endometroid adenocarcinoma (R0). The patient reports a single episode of cellulitis in the left leg, which was treated with antibiotics and compression therapy. She also reports recurrent pain and reduced range of motion. Indocyanine Green Lymphography shows linear and splash patterns in two regions of the leg with patent lymphatic vessels.”

*ChatGPT Treatment Recommendation: (18 Seconds)*

**Diagnosis:** Secondary (acquired) lymphedema of the left lower extremity, likely Stage II, following pelvic and paraaortal lymph node dissection for endometrial cancer.

**Rationale**:

The patient’s history of oncologic pelvic surgery, persistent unilateral limb swelling, and positive indocyanine green (ICG) lymphography findings are consistent with lymphedema. Lymph node dissection impairs normal lymphatic drainage, often resulting in fluid accumulation that cannot be resolved by simple elevation. The noted "linear and splash" patterns on ICG lymphography indicate partial lymphatic compromise with some lymphatic channels still patent but functioning sub-optimally.

**Recommended Evidence-Based Treatment Plan:**

1. Complete Decongestive Therapy (CDT): The cornerstone of lymphedema management includes:
   - Manual Lymphatic Drainage (MLD): Gentle, specialized massage techniques to promote lymph flow.
   - Compression Therapy: Use of compression bandaging followed by long-term compression garments to support tissue pressure and prevent fluid re-accumulation.
   - Skin Care: Meticulous hygiene and moisturization to prevent infections such as cellulitis.
   - Exercises: Targeted exercises to enhance lymphatic return and maintain range of motion.
2. Intermittent Pneumatic Compression (IPC): May be employed adjunctively, using sequential compression devices to further facilitate lymphatic drainage.
3. Consideration of Microsurgical Interventions: If conservative measures are insufficient, referral for a lymphaticovenular anastomosis (LVA) or lymph node transfer can be considered, given that patent lymphatic vessels are present. Such procedures can help restore or bypass obstructed channels.

**Benefits and Risks:**

- Benefits:
  - Volume Reduction and Symptom Relief: CDT can significantly reduce limb volume and discomfort, improving mobility and quality of life.
  - Decreased Infection Risk: Proper compression and skin care lower the incidence of cellulitis.
  - Long-Term Maintenance: When combined with ongoing self-management, CDT helps maintain stability in limb circumference and reduce pain.
- Risks and Considerations:
  - Skin Irritation or Discomfort: Compression garments and bandaging can sometimes cause skin irritation or discomfort.
  - Resource-Intensive Therapy: Regular MLD sessions and compression garment fittings require ongoing healthcare support and patient adherence.
  - Surgical Risks (if pursued): Microsurgical procedures carry operative and anesthesia-related risks, though they may yield longer-term improvements.

Overall, CDT remains the first-line, evidence-based approach. If the patient remains symptomatic despite optimal conservative management, evaluation by a specialist in lymphatic surgery is warranted.

***Case 6: Advanced Lower Extremity Lymphedema***

“A 50-year-old female presents with an eight-year history of severe, constant non-pitting swelling of the left foot and leg (38.5 cm vs. 36.9 cm), with fibrosis and hyperkeratosis. The patient has a history of bilateral serous ovarian carcinoma treated with hysterectomy, bilateral adnexectomy, omentectomy, appendectomy, cholecystectomy and pelvic and paraaortal lymphadenectomy (R0) and 6 cycles of carboplatin and taxol. The patient reports constant pain, reduced range of motion, and recurrent erysipelas and cellulitis, which have required repeated courses of antibiotics. Compression garments and manual lymphatic drainage have provided only minimal short-term relief. Indocyanine Green Lymphography shows a stardust pattern indicating lymphatic scarring.”

*ChatGPT Treatment Recommendation: (22 Seconds)*

**Diagnosis:** Advanced (Stage III) secondary lower-extremity lymphedema.

**Recommended Treatment Plan:**

Proceed with surgical intervention—specifically, circumferential suction-assisted lipectomy (liposuction)—in combination with ongoing comprehensive lymphedema therapy, including continuous compression garments and physical therapy. Microsurgical procedures, such as lymphaticovenular anastomosis (LVA) or vascularized lymph node transfer (VLNT), may also be considered if suitable, but liposuction is more reliably effective for advanced fibrotic lymphedema.

**Rationale:**

In this patient, long-standing (eight-year) secondary lymphedema, likely resulting from extensive pelvic lymph node dissection and subsequent scarring, has led to progressive fibrosis, hyperkeratosis, and non-pitting edema. Indocyanine green lymphography showing a “stardust” pattern signifies diffuse lymphatic dysfunction and compromised drainage. Non-invasive measures (compression therapy and manual lymphatic drainage) have only provided transient relief.

*Liposuction* directly removes excess fibroadipose tissue that has accumulated due to chronic lymph stasis. This surgical approach can significantly decrease limb volume, improve pain, restore range of motion, and reduce the frequency of infections like erysipelas and cellulitis. Although *LVA* or *VLNT* can help restore some physiologic lymphatic flow and potentially improve outcomes, these microsurgical techniques are technically challenging and better suited to earlier disease stages.

**Benefits and Risks:**

- Benefits:
  - Marked and durable reduction in limb volume and girth.
  - Improved mobility and quality of life.
  - Decreased risk of recurrent cellulitis and erysipelas.
  - Enhanced efficacy of compression therapy post-operatively.
- Risks:
  - Standard surgical risks (bleeding, infection, scarring).
  - Necessity of lifelong adherence to compression therapy and follow-up care to maintain results.
  - Potential for only partial restoration of normal limb contour and function, given the chronicity and severity of the disease.

Overall, liposuction combined with steadfast postoperative compression and possible adjunctive microsurgical procedures represents the most evidence-based, effective approach for advanced lymphedema that has not responded to conservative therapy.
