# Supplementary material for: Exploring the Role of AI in Managing Treatment Recommendations for Lymphedema: International, Multidisciplinary, Multiprofessional Survey Study of Trust, Reliability, and Impact on Decision-Making
Source: JMIR Med Inform. 2026 Apr 8;14:e80553. doi: 10.2196/80553 (PMC13060743; doi:10.2196/80553)
Supplement: Multimedia Appendix 2 [file medinform-v14-e80553-s002.docx]

**Supplementary Material 2.** Questionnaire with modified DISCERN tool

| Q1: The scenario was adequately analyzed, and an accurate diagnosis (including correct staging) was provided. (1-9) |
| --- |
| Q2: Is it relevant? (1-9)  (Hint. Are the provided suggestions relevant to the case study regarding lymphedema?) |
| Q3: Is it clear what sources of information were used to compile the information (1-9) ? |
| Q4: Is it clear when the information used was produced? (1-9)  (Hint: Is there any indication of how current the information is?) |
| Q5: Is it balanced and unbiased? (1-9) |
| Q6: Does it provide details of additional sources of support and information? (1-9) |
| Q7: Does it refer to areas of uncertainty? (1-9) |
| Q8: Does it describe how each treatment works? (1-9) |
| Q9: Does it describe the benefits of each treatment? (1-9) |
| Q10: Does it describe the risks of each treatment? (1-9) |
| Q11: Does it describe what would happen if no treatment is used? (1-9) |
| Q12: Does it describe how the treatment choices affect overall quality of life? (1-9) |
| Q13: Is it clear that there may be more than one possible treatment choice? (1-9) |
| Q14: Does Chat-GPT provide support for shared decision-making? (1-9) |
| Q15: Assess the overall quality of ChatGPT's response as a source of information regarding treatment options. (1-9) |
| Q16: I agree with the final recommended treatment option. (1-9) |

*Concluding Questions:*

| To what extent do you trust the generated responses as a reliable source of information for medical therapy suggestions? (1-9) |
| --- |
| Based on this study, to what extent would you consider using Artificial Intelligence such as ChatGPT in the future to assist in developing therapy suggestions for lymphedema. (1-9) |
| Would you recommend using ChatGPT as a supplementary tool for clinical decision-making? (1-9) |
| *Do you have any Comments, Observations & Suggestions:* |
