# Supplementary material for: Exploring the Role of AI in Managing Treatment Recommendations for Lymphedema: International, Multidisciplinary, Multiprofessional Survey Study of Trust, Reliability, and Impact on Decision-Making
Source: JMIR Med Inform. 2026 Apr 8;14:e80553. doi: 10.2196/80553 (PMC13060743; doi:10.2196/80553)
Supplement: Multimedia Appendix 3 [file medinform-v14-e80553-s003.docx]

**Supplementary Material 3.** Anonymous survey comments per subgroup

*Note: Minor spelling and typographical errors were corrected without altering the meaning or intent of the original participant comments.*

*Resident doctors:*

| Interesting, that ChatGPT specifically recommends compression garments during the day in many cases. You could also wear them at night. Additionally, I feel like it likes to tend to the conservative management side: e.g. always start with compression garments and MLD, although LVA or VLNT would clearly be indicated. |
| --- |
| Even though the information seems well organized, citations are usually missing which then affects the reliability for me. |
| ChatGPT writes long texts, in clinical practice I would want something shorter |
| To this date, 1.2.25, I would not use AI as a clinical decision-making tool without cross checking the information. But I would use it, when I have no idea how to treat a patient to receive a general direction what a should look for. |
| Overall, I think there is room for improvement to make an AI tool that is clinically and scientifically reliable and evidence based, but it has a lot of potential, and I am looking forward to implementing it in the clinical practice. |
| Using the right key words when asking ChatGPT, it can answer with a thorough rationale. |
| I think artificial Intelligence should (and already is being) be used for clinical reasoning. Not as a main decision maker, but to help clinicians, because it collects a wide amount of information. Sadly, sometimes also biased information and without any source-information |

*Board-certified doctors:*

| The exploration of surgical options is insufficient and there is an absence of indication as to how the information is compiled |
| --- |
| I think it may be part of the future work, but I think it needs further work in order to not only be a collection of "rumors/popular text " etc. |
| ChatGPT may provide useful information to a primary care team but should also place more emphasis on when to refer to lymphedema treatment team for further evaluation. |
| It does not seem to take most recent studies into account. Manual lymphatic drainage is not effective on its own. Prophylactic antibiotics are not discussed. Additional information from scans and biopsies are crucial from treatment. So is obesity management and compression therapy. |
| It can be useful for patients and people with no knowledge of lymphedema, but not for professionals. The evidence showed here is not based on the articles published, for example, CDT does not have any evidence, since there are not RCT available for CDT and lymphedema. |
| AI should be used as a source of information but not yet for clinical decisions |
| Surgical options are not well described or considered |
| It became clear that surgical information is not yet accessible enough, as information regarding surgical options was scarce. |
| Thoroughly impressed. I did notice that the circumference difference was not quite accurate with the severity though, and it lacked a couple of sentences about how LVA and lymph node transfer work. It did not include the concept of LyFT/LYST lymphatic system transfer (which is perhaps not that relevant yet in current practice in many places). Very well structured, worded and concise. Pictures did not correlate with circumference differences. Makes me consider using AI more in preparing articles, but important to have the citations/sources to be able to do quality control if the reader is unsure about the reliability of the content. |

*Physio / APNs:*

| In the case descriptions the circumferential measurements don't fit to the pictures, which could have influenced the information for ChatGPT. In addition, the recommendations are too general. In my experience, more individualized recommendations need to be made for a treatment plan to do justice to patients. There is too little information regarding exercises intensity. |
| --- |
| I always trust my hands and my feelings. I also believe in what the patient's body tells me. I consider not only the diagnosis, therapy, and medications, but also the patient's lifestyle, energy levels, and psychological state. ChatGPT is helpful, but individualized therapy is a professional opinion of a specialist based on experience. |
| Hello. Completing these questions was very interesting - on one hand it can be helpful to find ChatGPT responses like this, on the other hand each situation is unique, and I think that it should not replace our clinical reasoning. |
