# Supplementary material for: Exploring the Role of AI in Managing Treatment Recommendations for Lymphedema: International, Multidisciplinary, Multiprofessional Survey Study of Trust, Reliability, and Impact on Decision-Making
Source: JMIR Med Inform. 2026 Apr 8;14:e80553. doi: 10.2196/80553 (PMC13060743; doi:10.2196/80553)
Supplement: Multimedia Appendix 4 [file medinform-v14-e80553-s004.docx]

**Supplementary Material 4.** Group comparisons for questionnaire responses divided by case

**Case 1**

|  | **Overall** | **Resident Doctor** | **Board-Certified Doctor** | **Physio / APN** | **F (df1, df2)** | **Levene’s Test for Equality of Variables** | **p- value** |
| --- | --- | --- | --- | --- | --- | --- | --- |
| **Q1** | 7.8 (±1.1) | 8.0 (±0.8) | 7.2 (±1.5) | 8.2 (±0.6) | 4.8 (2, 63.0) | 0.063 | *0.011* |
| **Q2** | 7.8 (±1.2) | 8.2 (±0.8) | 7.4 (±1.4) | 7.6 (±1.6) | 3.3 (2, 60.0) | 0.403 | *0.043* |
| **Q3** | 3.3 (±2.5) | 2.6 (±2.2) | 4.2 (±2.5) | 3.4 (±3.2) | 2.8 (2, 63.0) | 0.212 | 0.066 |
| **Q4** | 2.9 (±2.3) | 2.2 (±1.6) | 3.9 (±2.6) | 3.0 (±2.9) | 3.9 (2, 62.0) | 0.058 | *0.024* |
| **Q5** | 6.3 (±1.7) | 6.3 (±1.7) | 6.4 (±1.4) | 5.7 (±2.3) | 0.6 (2, 62.0) | 0.433 | 0.552 |
| **Q6** | 3.4 (±2.5) | 2.8 (±2.3) | 4.3 (±2.4) | 3.4 (±2.8) | 2.4 (2, 63.0) | 0.709 | 0.096 |
| **Q7** | 3.7 (±2.2) | 3.1 (±2.0) | 4.1 (±1.8) | 4.9 (±2.8) | 3.7 (2, 63.0) | 0.265 | *0.031* |
| **Q8** | 6.7 (±2.0) | 7.0 (±2.1) | 6.5 (±1.8) | 6.0 (±2.1) | 1.2 (2, 63.0) | 0.991 | 0.320 |
| **Q9** | 7.0 (±1.6) | 7.3 (±1.7) | 6.9 (±1.2) | 6.4 (±2.0) | 1.2 (2, 62.0) | 0.304 | 0.299 |
| **Q10** | 6.3 (±2.1) | 6.2 (±2.4) | 6.3 (±1.7) | 6.5 (±1.7) | 0.1 (2, 63.0) | 0.318 | 0.928 |
| **Q11** | 3.1 (±2.4) | 2.3 (±2.1) | 3.9 (±2.2) | 3.9 (±3.1) | 3.7 (2, 63.0) | 0.163 | *0.030* |
| **Q12** | 4.6 (±2.4) | 4.3 (±2.7) | 4.7 (±2.2) | 5.4 (±3.2) | 0.8 (2, 62.0) | 0.348 | 0.450 |
| **Q13** | 6.7 (±2.3) | 7.7 (±1.7) | 5.9 (±2.1) | 5.4 (±3.2) | 6.7 (2, 21.3) | *0.004* | *0.005* |
| **Q14** | 4.9 (±2.4) | 4.9 (±2.6) | 5.0 (±2.1) | 5.1 (±2.5) | 0.1 (2, 63.0) | 0.504 | 0.968 |
| **Q15** | 6.6 (±1.4) | 7.1 (± 1.1) | 6.4 (±1.3) | 5.7 (±2.0) | 5.2 (2, 63.0) | 0.172 | *0.009* |
| **Q16** | 7.4 (±1.7) | 8.0 (±0.9) | 7.2 (±1.5) | 6.1 (±2.9) | 3.9 (2, 19.2) | *0.004* | *0.036* |

Values are depicted mean (± standard deviation), unless reported otherwise. *Values in italic demonstrate statistical significance (p<0.05).*

**Case 2**

|  | **Overall** | **Resident Doctor** | **Board-Certified Doctor** | **Physio / APN** | **F (df1, df2)** | **Levene’s Test for Equality of Variables** | **p- value** |
| --- | --- | --- | --- | --- | --- | --- | --- |
| **Q1** | 6.9 (±1.9( | 7.2 (±1.6) | 6.3 (±2.1) | 7.0 (±2.3) | 1.9 (2, 63.0) | 0.936 | 0.156 |
| **Q2** | 7.5 (±1.3) | 7.9 (±1.0) | 6.9 (±1.5) | 7.7 (±1,0) | 4.7 (2, 61.0) | 0.166 | *0.013* |
| **Q3** | 3.1 (±2.3) | 2.4 (±2.1) | 4.0 (±2.2) | 3.5 (±2.7) | 3.5 (2, 62.0) | 0.315 | *0.035* |
| **Q4** | 2.7 (±2.1) | 2.1 (±1.8) | 3.4 (±2.1) | 3.2 (±2.9) | 3.0 (2, 62.0) | 0.200 | 0.055 |
| **Q5** | 6.0 (±1.6) | 6.2 (±1.6) | 5.7 (±1.4) | 6.0 (±2.1) | 0.6 (2, 63.0) | 0.827 | 0.561 |
| **Q6** | 3.6 (±2.3) | 2.9 (±2.3) | 4.4 (±1.9) | 4.0 (±2.7) | 3.2 (2, 63.0) | 0.435 | *0.049* |
| **Q7** | 4.5 (±2.0) | 3.8 (±2.0) | 5.4 (±1.5) | 4.7 (±2.5) | 4.8 (2, 62.0) | 0.352 | *0.011* |
| **Q8** | 5.9 (±2.1) | 6.1 (±2.2) | 5.8 (±1.9) | 5.5 (±2.2) | 0.3 (2, 63.0) | 0.775 | 0.716 |
| **Q9** | 7.1 (±1.4) | 7.3 (±1.5) | 6.7 (±1.4) | 7.0 (±1.1) | 1.1 (2, 63.0) | 0.793 | 0.341 |
| **Q10** | 6.9 (±1.6) | 7.3 (±1.6) | 6.9 (±13) | 5.9 (±2.1) | 2.8 (2, 62.0) | 0.353 | 0.068 |
| **Q11** | 3.4 (±2.1) | 2.7 (±1.8) | 4.2 (±2.1) | 3.9 (±2.6) | 4.1 (2, 60.0) | 0.239 | *0.022* |
| **Q12** | 4.6 (±2.3) | 4.3 (±2.4) | 4.7 (±2.1) | 5.4 (±2.6) | 1.0 (2, 63.0) | 0.743 | 0.376 |
| **Q13** | 7.4 (±1.6) | 8.1 (±1.1) | 6.6 (±1.6) | 7.2 (±1.8) | 7.2 (2, 63.0) | 0.534 | *0.001* |
| **Q14** | 5.8 (±2.1) | 5.8 (±2.2) | 5.8 (±2.0) | 5.8 (±2.4) | 0.0 (2, 63.0) | 0.780 | 0.998 |
| **Q15** | 6.8 (±1.4) | 7.1 (±1.1) | 6.7 (±1.5) | 6.0 (±1.9) | 2.7 (2, 63.0) | 0.187 | 0.073 |
| **Q16** | 7.1 (±1.4) | 7.4 (±1.3) | 6.8 (±1.6) | 7.1 (±1.4) | 1.3 (2, 63.0) | 0.934 | 0.288 |

Values are depicted mean (± standard deviation), unless reported otherwise. *Values in italic demonstrate statistical significance (p<0.05).*

**Case 3**

|  | **Overall** | **Resident Doctor** | **Board-Certified Doctor** | **Physio / APN** | **F (df1, df2)** | **Levene’s Test for Equality of Variables** | **p- value** |
| --- | --- | --- | --- | --- | --- | --- | --- |
| **Q1** | 7.4 (±1.4) | 7.7 (±1.1) | 6.8 (±1.3) | 7.3 (±2.4) | 2.8 (2, 63.0) | 0.345 | 0.068 |
| **Q2** | 7.3 (±1.4) | 7.6 (±1.2) | 6.7 (±1.7) | 7.5 (±1.0) | 3.0 (2, 63.0) | 0.507 | 0.056 |
| **Q3** | 3.0 (±2.4) | 2.3 (±2.1) | 3.8 (±2.4) | 3.7 (±3.0) | 3.5 (2, 63.0) | 0.090 | *0.036* |
| **Q4** | 2.6 (±2.0) | 1.8 (±1.2) | 3.4 (±2.1) | 3.1 (±2.9) | 6.5 (2, 20.0) | *0.029* | *0.007* |
| **Q5** | 5.6 (±1.9) | 5.9 (±1.9) | 5.3 (±1.7) | 5.4 (±2.3) | 0.8 (2, 63.0) | 0.712 | 0.469 |
| **Q6** | 3.5 (±2.2) | 3.0 (±2.1) | 4.3 (±1.9) | 3.6 (±2.6) | 2.7 (2, 63.0) | 0.291 | 0.075 |
| **Q7** | 4.4 (±2.1) | 4.1 (±2.1) | 5.1 (±1.8) | 4.1 (±2.5) | 1.6 (2, 61.0) | 0.215 | 0.209 |
| **Q8** | 6.5 (±1.8) | 6.5 (±2.1) | 6.4 (±1.2) | 6.4 (±19) | 0.02 (2, 21.4) | *0.037* | 0.978 |
| **Q9** | 6.9 (±1.8) | 7.0 (±2.0) | 6.7 (±1.3) | 6.7 (±2.0) | 0.2 (2, 63.0) | 0.448 | 0.792 |
| **Q10** | 6.6 (±1.9) | 6.8 (±2.2) | 6.6 (±1.4) | 6.0 (±2.3) | 0.7 (2, 63.0) | 0.326 | 0.513 |
| **Q11** | 3.6 (±2.3) | 2.8 (±2.1) | 4.8 (±1.9) | 3.9 (±2.9) | 5.8 (2. 62.0) | 0.142 | *0.005* |
| **Q12** | 4.8 (±2.4) | 4.6 (±2.4) | 5.1 (±2.2) | 4.9 (±3.0) | 0.4 (2, 63.0) | 0.465 | 0.667 |
| **Q13** | 6.8 (±1.8) | 7.3 (±1.8) | 6.5 (±1.6) | 5.8 (±1.8) | 3.3 (2, 63.0) | 0.721 | *0.045* |
| **Q14** | 5.3 (±2.3) | 5.2 (±2.5) | 5.6 (±2.0) | 5.0 (±2.3) | 0.3 (2. 63.0) | 0.501 | 0.782 |
| **Q15** | 6.2 (±1.9) | 6.6 (±1.7) | 5.8 (±2.2) | 5.6 (±1.7) | 1.9 (2, 63.0) | 0.337 | 0.158 |
| **Q16** | 6.1 (±2.2) | 6.6 (±1.8) | 5.9 (±2.4) | 4.9 (±2.6) | 2.7 (2, 63.0) | 0.246 | 0.077 |

Values are depicted mean (± standard deviation), unless reported otherwise. *Values in italic demonstrate statistical significance (p<0.05).*

**Case 4**

|  | **Overall** | **Resident Doctor** | **Board-Certified Doctor** | **Physio / APN** | **F (df1, df2)** | **Levene’s Test for Equality of Variables** | **p- value** |
| --- | --- | --- | --- | --- | --- | --- | --- |
| **Q1** | 7.4 (±1.5) | 7.9 (±1.2) | 6.5 (±1.6) | 8.1 (±1.0) | 8.3 (2, 63.0) | 0.288 | *0.001* |
| **Q2** | 7.5 (±1.5) | 8.0 (±1.0) | 6.6 (±1.8) | 7.9 (±1.0) | 5.5 (2, 23.9) | *0.038* | *0.011* |
| **Q3** | 3.2 (±2.5) | 2.6 (±2.2) | 3.8 (±2.5) | 3.9 (±3.0) | 2.3 (2, 63.0) | 0.164 | 0.111 |
| **Q4** | 2.7 (±2.1) | 2.0 (±1.6) | 3.4 (±2.1) | 3.3 (±3.0) | 3.7 (2, 63.0) | 0.097 | *0.030* |
| **Q5** | 5.8 (±1.9) | 6.1 (±1.9) | 5.5 (±1.7) | 5.3 (±2.5) | 0.9 (2, 63.0) | 0.807 | 0.424 |
| **Q6** | 3.6 (±2.3) | 3.0 (±2.1) | 4.3 (±2.0) | 3.9 (±2.9) | 2.4 (2, 63.0) | 0.219 | 0.098 |
| **Q7** | 3.9 (±2.0) | 3.5 (±2.0) | 4.4 (±1.6) | 4.3 (±2.8) | 1.4 (2, 63.0) | 0.084 | 0.255 |
| **Q8** | 6.6 (±1.6) | 6.8 (±1.8) | 6.5 (±1.2) | 6.0 (±1.9) | 1.0 (2, 62.0) | 0.281 | 0.393 |
| **Q9** | 7.0 (±1.5) | 7.4 (±1.7) | 6.6 (±1.2) | 6.8 (±1.1) | 2.0 (2, 61.0) | 0.507 | 0.145 |
| **Q10** | 6.6 (±1.8) | 6.8 (±2.0) | 6.5 (±1.3) | 6.4 (±2.2) | 0.4 (2, 63.0) | 0.249 | 0.709 |
| **Q11** | 3.6 (±2.2) | 2.8 (±1.9) | 4.4 (±1.8) | 4.7 (±2.8) | 5.6 (2, 63.0) | 0.392 | *0.006* |
| **Q12** | 4.8 (±2.3) | 4.4 (±2.4) | 5.2 (±2.0) | 5.2 (±2.9) | 1.1 (2, 62.0) | 0.356 | 0.337 |
| **Q13** | 6.5 (±2.0) | 7.1 (±2.1) | 5.9 (±1.8) | 6.3 (±1.9) | 2.5 (2, 63.0) | 0.799 | 0.092 |
| **Q14** | 5.3 (±2.3) | 5.2 (±2.5) | 5.6 (±2.1) | 5.2 (±2.2) | 0.2 (2, 63.0) | 0.559 | 0.793 |
| **Q15** | 6.7 (±1.5) | 7.1 (±1.1) | 6.1 (±19) | 6.5 (±1.4) | 3.0 (2, 63.0) | 0.082 | 0.058 |

Values are depicted mean (± standard deviation), unless reported otherwise. *Values in italic demonstrate statistical significance (p<0.05).*

**Case 5**

|  | **Overall** | **Resident Doctor** | **Board-Certified Doctor** | **Physio / APN** | **F (df1, df2)** | **Levene’s Test for Equality of Variables** | **p- value** |
| --- | --- | --- | --- | --- | --- | --- | --- |
| **Q1** | 7.2 (±1.5) | 7.4 (±1.3) | 7.0 (±1.4) | 7.0 (±2.1) | 0.5 (2, 63.0) | 0.632 | 0.605 |
| **Q2** | 7.4 (±1.3) | 7.7 (±1.1) | 6.9 (±1.3) | 7.4 (±1.3) | 3.4 (2, 63.0) | 0.790 | *0.041* |
| **Q3** | 3.3 (±2.4) | 2.6 (±2.1) | 4.2 (±2.3) | 4.0 (±3.1) | 4.1 (2, 22.2) | *0.035* | *0.031* |
| **Q4** | 2.8 (±2.0) | 2.2 (±1.5) | 3.4 (±2.0) | 3.3 (±2.9) | 3.2 (2, 63.0) | 0.074 | *0.047* |
| **Q5** | 5.6 (±1.7) | 5.8 (±1.6) | 5.5 (±1.6) | 5.3 (±2.0) | 0.4 (2, 63.0) | 0.990 | 0.654 |
| **Q6** | 3.6 (±2.0) | 3.2 (±2.1) | 4.0 (±1.7) | 3.9 (±2.6) | 1.4 (2, 63.0) | 0.150 | 0.264 |
| **Q7** | 4.2 (±2.0) | 3.8 (±2.1) | 4.7 (±1.7) | 4.7 (±2.5) | 1.8 (2, 63.0) | 0.737 | 0.176 |
| **Q8** | 6.3 (±1.6) | 6.4 (±1.8) | 6.1 (±1.4) | 6.7 (±1.0) | 0.7 (2, 62.0) | 0.112 | 0.502 |
| **Q9** | 6.7 (±1.5) | 6.7 (±1.8) | 6.6 (±1.2) | 6.9 (±1.2) | 0.2 (2, 63.0) | 0.380 | 0.848 |
| **Q10** | 6.6 (±1.7) | 6.7 (±1.9) | 6.4 (±1.3) | 6.8 (±1.7) | 0.2 (2, 63.0) | 0.42 | 0.796 |
| **Q11** | 3.5 (±2.2) | 2.9 (±2.2) | 4.2 (±1.8) | 4.0 (±2.8) | 2.8 (2, 63.0) | 0.186 | 0.070 |
| **Q12** | 4.9 (±2.4) | 4.6 (±2.4) | 5.0 (±2.3) | 5.3 (±3.1) | 0.4 (2, 63.0) | 0.828 | 0.691 |
| **Q13** | 7.0 (±1.4) | 7.4 (±1.5) | 6.7 (±1.4) | 6.4 (±1.4) | 2.4 (2, 63.0) | 0.751 | 0.101 |
| **Q14** | 5.3 (±2.2) | 5.2 (±2.3) | 5.7 (±2.0) | 5.0 (±2.3) | 0.5 (2, 63.0) | 0.596 | 0.607 |
| **Q15** | 6.4 (±1.6) | 6.8 (±1.3) | 6.1 (±1.8) | 6.1 (±1.8) | 1.7 (2, 63.0) | 0.336 | 0.199 |
| **Q16** | 6.7 (±1.8) | 6.7 (±1.9) | 6.4 (±1.7) | 6.9 (±2.1) | 0.3 (2, 63.0) | 0.850 | 0.757 |

Values are depicted mean (± standard deviation), unless reported otherwise. *Values in italic demonstrate statistical significance (p<0.05).*

**Case 6**

|  | **Overall** | **Resident Doctor** | **Board-Certified Doctor** | **Physio / APN** | **F (df1, df2)** | **Levene’s Test for Equality of Variables** | **p- value** |
| --- | --- | --- | --- | --- | --- | --- | --- |
| **Q1** | 7.4 (±1.5) | 7.9 (±1.0) | 6.8 (±1.4) | 7.2 (±2.4) | 4.0 (2, 62.0) | 0.198 | *0.023* |
| **Q2** | 7.2 (±1.5) | 7.7 (±1.1) | 6.4 (±1.8) | 7.6 (±1.1) | 6.3 (2, 63.0) | 0.265 | *0.003* |
| **Q3** | 3.3 (±2.6) | 2.5 (±2.2) | 4.1 (±2.6) | 4.2 (±3.4) | 3.3 (2, 62.0) | 0.114 | *0.042* |
| **Q4** | 2.8 (±2.2) | 2.2 (±1.7) | 3.4 (±2.1) | 3.6 (±3.3) | 3.3 (2, 63.0) | 0.101 | *0.044* |
| **Q5** | 5.5 (±1.8) | 5.7 (±1.7) | 5.5 (±1.8) | 5.2 (±2.4) | 0.3 (2, 63.0) | 0.811 | 0.774 |
| **Q6** | 3.4 (±2.3) | 2.7 (±2.0) | 4.1 (±2.2) | 4.2 (±3.1) | 3.7 (2, 63.0) | 0.200 | *0.029* |
| **Q7** | 4.2 (±2.1) | 4.0 (±2.1) | 4.3 (±2.0) | 4.5 (±2.8) | 0.3 (2, 63.0) | 0.534 | 0.759 |
| **Q8** | 6.1 (±1.9) | 6.2 (±2.0) | 6.2 (±1.5) | 5.7 (±2.6) | 0.2 (2, 63.0) | 0.300 | 0.783 |
| **Q9** | 6.5 (±1.9) | 6.7 (±1.9) | 6.4 (±1.4) | 5.8 (±2.4) | 0.9 (2, 63.0) | 0.285 | 0.407 |
| **Q10** | 6.3 (±2.0) | 6.6 (±2.1) | 6.0 (±1.8) | 6.0 (±2.3) | 0.6 (2, 63.0) | 0.645 | 0.582 |
| **Q11** | 3.7 (±2.2) | 3.2 (±2.0) | 4.3 (±2.1) | 4.0 (±2.9) | 1.8 (2, 61.0) | 0.202 | 0.171 |
| **Q12** | 4.6 (±2.3) | 4.4 (±2.3) | 4.7 (±2.3) | 5.2 (±2.9) | 0.5 (2, 62.0) | 0.847 | 0.608 |
| **Q13** | 6.4 (±2.1) | 7.0 (±1.9) | 6.3 (±1.9) | 5.0 (±2.4) | 3.9 (2, 63.0) | 0.592 | *0.025* |
| **Q14** | 5.2 (±2.4) | 5.1 (±2.3) | 5.5 (±2.3) | 4.4 (±2.8) | 0.8 (2, 63.0) | 0.801 | 0.462 |
| **Q15** | 6.4 (±1.8) | 6.6 (±1.6) | 6.1 (±2.0) | 6.1 (±2.3) | 0.5 (2, 63.0) | 0.464 | 0.609 |
| **Q16** | 6.6 (±2.0) | 6.9 (±1.7) | 6.4 (±2.3) | 6.0 (±2.5) | 1.0 (2, 63.0) | 0.476 | 0.366 |

Values are depicted mean (± standard deviation), unless reported otherwise. *Values in italic demonstrate statistical significance (p<0.05).*

**Concluding Questions**

|  | **Overall** | **Resident Doctor** | **Board-Certified Doctor** | **Physio / APN** | **F (df1, df2)** | **Levene’s Test for Equality of Variables** | **p- value** |
| --- | --- | --- | --- | --- | --- | --- | --- |
| **Q1** | 5.9 (±1.6) | 6.5 (±1.0) | 5.4 (±1.9) | 5.1 (±1.8) | 5.2 (2, 20.6) | *0.027* | *0.015* |
| **Q2** | 5.9 (±1.9) | 6.5 (±1.4) | 5.3 (±2.2) | 5.3 (±2.3) | 3.2 (2, 63.0) | 0.119 | *0.047* |
| **Q3** | 6.1 (±2.1) | 7.0 (±1.4) | 5.4 (±2.4) | 4.7 (±2.0) | 8.1 (2, 21.6) | *0.015* | *<0.001* |

Values are depicted mean (± standard deviation), unless reported otherwise. *Values in italic demonstrate statistical significance (p<0.05).*
